# Community pharmacists’ comfort levels with and barriers to application of an expanded scope of practice in Québec

**DOI:** 10.1177/17151635241264517

**Published:** 2024-07-30

**Authors:** Léonie Rouleau, Léa Prince-Duthel, Marie-Claude Vanier, Nicolas Dugré, Anne Maheu, Line Guénette

## Abstract

**Background:**

In recent years, community pharmacists have seen their profession transition from a dispensing-focused role to a rapidly evolving clinically oriented practice. In Québec, Bill 31, adopted in 2020, increased the clinical opportunities for pharmacists with independent prescribing privileges in various defined clinical situations. As this expanded role can lead to different barriers, it is crucial to explore pharmacists’ comfort levels with implementing such changes in their practice.

**Methods:**

A web-based survey was conducted from March 25 to May 28, 2021, among community pharmacists in Québec. We collected data with a questionnaire developed for this study. Questions were grouped into 4 domains: (1) characteristics of the respondents; (2) workload and work setting; (3) comfort level with, and barriers to, adjusting medications and following up pharmacologic treatments (86 clinical situations evaluated); and (4) general barriers and facilitators to implementation.

**Results:**

A total of 146 community pharmacists completed the questionnaire. Most were women (71.9%), younger than 50 years of age (86.2%), had a bachelor’s degree (64.4%) as their highest academic level and had more than 10 years of experience as pharmacists (56.8%). Most of them worked exclusively in a community pharmacy (86.3%). Among the 86 clinical situations evaluated, there were 16 in which at least 80% of respondents felt comfortable. The main barriers identified were a lack of knowledge, experience and dedicated time and difficulties integrating these activities into the workflow; facilitators were having an adequate environment and resources.

**Conclusion:**

This study shows community pharmacists can confidently adjust pharmacotherapy for several conditions. However, they must have adequate time and resources. Also, the more complex the clinical situations were, the less comfortable community pharmacists felt adjusting pharmacotherapy. This study identified several areas where continuing education, training and mentoring could be offered and where the work environment and organization could be improved.

**Knowledge into Practice**

- Following their expanded scope of practice in March 2020, community pharmacists in Québec are prepared to adjust pharmacotherapy and ensure follow-ups in several clinical situations.
- Conditions for which pharmacists generally felt less comfortable managing pharmacotherapy included bipolar disorder, major neurocognitive disorder, behavioural and psychological symptoms of dementia, COPD and asthma.
- Continuing education, training and mentoring by experienced pharmacists could be offered since the main barriers were a lack of experience and knowledge.
- Adjustments to the practice environment and work organization, such as dedicated areas and time slots, delegation of some activities to pharmacy technicians and practice tools could help pharmacists implement these new activities in their practice.

## Introduction

In recent years, community pharmacists have seen their profession transition from a traditional dispensing-focused role to a more clinically oriented practice. Canada is a leader in expanding pharmacists’ scope of practice and prescribing rights based on provincial regulations.^1^ In Alberta, pharmacists have been prescribing medications independently since 2007.^2^ In Québec, the first significant prescribing privileges for pharmacists came with Bill 41, which updated the Pharmacy Act, adopted in June 2015. It allowed pharmacists to extend prescriptions to avoid treatment discontinuation, prescribe medications for common ailments or when the diagnosis is already known (for a selection of diagnoses), adjust medications for safety reasons or to reach clinical targets and prescribe a limited number of laboratory tests for medication monitoring.^3^

In March 2020, Bill 31 was adopted, expanding pharmacists’ scope of practice further to facilitate access to primary care for patients.^4^ Pharmacists were granted more possibilities for independent prescribing, including any laboratory tests required to monitor the patient’s drug therapy, vaccines, completely autonomous medication adjustments (modification of pharmaceutical form, dosage or prescribed regimen) and initiating medication for additional situations (e.g., shingles, flu, contraception, Lyme disease prophylaxis).^4^ This expanded scope of practice increased pharmacists’ responsibility as independent prescribers, and many pharmacists had not been formally trained for this new role. Practice changes are often accompanied by periods of discomfort, can lead to various barriers and can bring new needs for those having to change.^5,6^ The level of confidence in and comfort with these new responsibilities depends on many factors and may vary among practitioners and according to contexts. A study performed in Alberta, shortly after pharmacists could initiate prescription medications independently,^6^ explored the factors influencing pharmacists’ adoption of new prescribing practices. Essentially, they found that knowledge, skills, confidence, motivation and adequate resources were key factors influencing pharmacists’ prescribing activities.^6^

**Mise En Pratique Des Connaissances**

- Suite à l’élargissement de leur champ d’activité adopté en mars 2020, les pharmaciens communautaires du Québec sont prêts à ajuster la pharmacothérapie et à assurer les suivis dans plusieurs situations cliniques.
- Les pharmaciens se sentent généralement moins à l’aise de gérer la pharmacothérapie en cas de trouble bipolaire, de trouble neurocognitif majeur, de symptômes comportementaux et psychologiques de la démence, de MPOC et d’asthme.
- L’éducation continue, la formation et le mentorat dispensés par des pharmaciens expérimentés pourraient être proposés, les principaux obstacles étant le manque d’expérience et de connaissances.
- Des aménagements de l’environnement et de l’organisation du travail, tels que des espaces et des créneaux horaires réservés, la délégation de certaines activités à des techniciens en pharmacie et des outils pratiques pourraient aider les pharmaciens à mettre en œuvre ces nouvelles activités dans leur pratique.

To our knowledge, there have been no published studies on how Québec community pharmacists have integrated these new prescribing activities into their daily work. Understanding their comfort with their expanded role and the barriers to implementing these activities is essential to reorganizing and providing pharmaceutical services and identifying continuing education needs. The Réseau Québécois des pharmaciens GMF (RQP GMF), a practice community of pharmacists working in family medicine groups, promotes and supports advanced clinical practice and professional collaboration.^7^ Knowing community pharmacists’ comfort with independent medication adjustment could help family medicine group pharmacists determine which types of adjustments and follow-ups could be transferred from primary care clinics to community pharmacists, to optimize practices and avoid duplication. The purpose of this study was to examine community pharmacists’ comfort levels with adjusting and managing pharmacotherapy in different clinical situations. We also identify potential barriers and facilitators to ensure optimal implementation of pharmacists’ expanded scope of practice.

## Methods

### Study design

A cross-sectional web-based survey was performed among pharmacists practising in the province of Québec.

### Recruitment

A link to the questionnaire was distributed through the newsletters of several provincial professional associations: Ordre des pharmaciens du Québec (OPQ), Association professionnelle des pharmaciens salariés du Québec (APPSQ), RQP GMF and Comité régional sur les services pharmaceutiques (CRSP). It was also shared on private Facebook groups of pharmacists.

### Data collection

The questionnaire was available from March 25 to May 28, 2021. It was developed for this study by the RQP GMF research group. Questions were partly based on previous surveys carried out by our team and related to the new activities allowed for pharmacists. It was pretested by collaborators, all pharmacists, to ensure that the questions were relevant and understandable and that the time required for completion was acceptable. The complete questionnaire, in French, is available as Appendix 1 in Supplemental Materials.

Survey questions explored 4 domains: (1) characteristics of pharmacists, (2) workload and work setting, (3) comfort level (5-point Likert scale) and barriers (6 predefined answers) regarding adjusting medications and following up pharmacologic treatments in 16 conditions and (4) general barriers and facilitators to implementing those activities.

The characteristics of pharmacists (8 questions) included their age, sex, academic degree, graduation year, years of experience in different settings, work title and work settings. The workload and characteristics of the work environment section (9 questions) included the hourly workload per pharmacist, the implementation of Tech-Check-Tech, allowing a technician to perform content medication verification that is prepared by another technician,^8^ time allocated to clinical follow-ups, interprofessional collaboration, number of pharmacists on the team, integration of vaccination and integration of activities previously granted (in 2015 by Bill 41).

Pharmacists’ comfort level with performing medication adjustments and follow-ups for 16 different conditions frequently encountered in primary care was assessed with a 5-point Likert scale (1 = *completely comfortable*, 2 = *fairly comfortable*, 3 = *moderately comfortable*, 4 = *not very comfortable*, 5 = *not at all comfortable*). Clinical conditions included in the survey were hypothyroidism, diabetes mellitus (DM) with or without insulin, hypertension, heart failure, dyslipidemia, anticoagulation therapy, migraine prophylaxis, depression, anxiety disorders, insomnia, chronic pain, major neurocognitive disorder and behavioural and psychological symptoms of dementia (BPSD), chronic obstructive pulmonary disease (COPD), asthma, attention-deficit hyperactivity disorder (ADHD) and bipolar disorder. These conditions were divided into various complexity levels according to presentation factors. For example, the different levels of complexity (less to most complex) for hypothyroidism were the following: stable hypothyroidism, newly diagnosed hypothyroidism, hypothyroidism in geriatrics, hypothyroidism in pregnancy, recent thyroid gland surgery and history of thyroid cancer. The survey represented a total of 86 clinical situations. For each clinical situation, we asked pharmacists to indicate the main obstacle to their feeling comfortable in follow-ups and adjustments. They had to choose among 6 predefined answers: no barrier, lack of clinical experience to perform the tasks with confidence, lack of knowledge on the subject, lack of accessibility to a professional to whom I can refer in case of questioning, lack of clear guidance from the prescriber (clinical targets, maximum doses and follow-up, expected communication) and lack of interest from the pharmacist.

Finally, pharmacists were asked to provide up to 3 general barriers to implementing their expanded scope of practice, and facilitators or solutions to support greater adoption of these new activities by pharmacists.

Respondents completing the questionnaire were deemed to have given consent. The study was approved by the Ethics Committee of Université de Montréal (#2018-669).

### Analysis

Although the questionnaire was also sent to family medicine group pharmacists, the analysis presented in this article is restricted to community pharmacists. We performed a descriptive analysis using Excel. First, we described the participants and their work setting. Second, to describe the comfort level with performing medication adjustments and follow-ups, we grouped the categories *totally* and *fairly comfortable* to form a *comfortable* category. We also grouped *not very* and *not at all comfortable* to form an *uncomfortable* category. We then separated situations based on the proportion of pharmacists declaring they were comfortable to reveal which clinical situations were mastered or not by the respondents: ≥80% (almost all respondents), >50% to <80% (most), ≥20% to <50% (some) and <20% (very few). For clinical situations with very few comfortable respondents, we looked more closely at what was mentioned as their main obstacle to feeling comfortable in following up and adjusting pharmacotherapy.

## Results

### Characteristics of respondents

Among the 7162 community pharmacists registered in Québec in March 2021, 300 started to fill out the questionnaire and 146 completed it. The results compiled in this study consider only the completed surveys. The characteristics of the participants are presented in Table 1. Most were women (71.9%), younger than 50 years of age (86.2%), had a bachelor’s degree (64.4%) as their highest level of education and had more than 10 years of experience as registered pharmacists (56.8%). Among the participants, 54.8% were full-time community pharmacists and 23.3% were pharmacy owners. Most of them worked exclusively in a community pharmacy (86.3%).

**Table 1:** Characteristics of community pharmacists (*N* = 146)

	Total (*N* = 146)	
Community pharmacist characteristic	*n*	%
Sex		
Female	105	71.9
Male	41	28.1
Age in years		
<30	32	21.9
30–39	50	34.2
40–49	44	30.1
50–59	15	10.3
≥60	5	3.4
Pharmacy degree		
Bachelor of pharmacy (PharmB)	94	64.4
Doctor of pharmacy (PharmD)	48	32.9
Certificate of qualification in pharmacy (QeP)	4	2.7
Experience as a licensed pharmacist		
None	1	0.7
<6 months	1	0.7
6–12 months	7	4.8
>1–2 years	11	7.5
>2–5 years	22	15.1
>5–10 years	21	14.4
>10 years	83	56.8
Work title		
Full-time community pharmacist	80	54.8
Part-time community pharmacist	22	15.1
Substitute pharmacist	7	4.8
Chief pharmacist	3	2.1
Community pharmacy owner	34	23.3
Other work setting (multiple answers possible)		
Yes, in health care facility (hospital setting)	5	3.4
Yes, in academic affiliation (university)	12	8.2
No, I only work in community pharmacy	126	86.3
Other	4	2.7

### Characteristics of work settings and practices

The work settings of community pharmacists were also characterized (Table 2). The hourly workload for prescriptions delivered was generally 20 to 40 prescriptions per working hour per pharmacist (64.3%). Tech-Check-Tech was implemented for medication served in blister packs for 48.6% of the respondents. Regarding the time allocated to ensure clinical follow-ups, only 6.2% of the respondents had a period allocated to this in their daily schedule. Most pharmacists (61%) estimated using 10% to 29% of their working time for clinical follow-ups.

**Table 2:** Workload and characteristics of the work environment (*N* = 146)

Characteristic	*n*	%
Hourly workload (prescriptions) per pharmacist		
50+	8	5.5
40–50	13	8.9
30–40	44	30.1
20–30	50	34.2
<20	15	10.3
I don’t know	16	11.0
Implementation of Tech-Check-Tech		
Pill dispensers and refill baskets	6	4.1
Pill dispensers	71	48.6
Partially or occasionally	10	6.8
No	59	40.4
Time allocated to ensure clinical follow-ups		
Yes, scheduled time slot each day	9	6.2
Yes, scheduled time slot on certain days	16	11.0
No dedicated time, but certain moments	60	41.1
None	61	41.8
Percentage of time reserved for clinical follow-ups (average: 13.8%)		
0	7	4.8
>0% to <10%	36	24.7
10% to <20%	56	38.4
20% to <30%	33	22.6
30% to <40%	9	6.2
≥40%	5	3.4
Interprofessional collaboration with professionals		
Great openness on both sides	27	18.5
Professionals available to answer calls from pharmacists	53	36.3
Difficulties with reaching physicians but some collaboration	63	43.2
No openness to collaboration	1	0.7
Other	2	1.4
Number of pharmacists on the team		
1–3	34	23.3
4–6	84	57.5
7 and more	28	19.2
Vaccination service offered		
No	15	10.3
Nurses	54	37.0
Pharmacists	33	22.6
Nurses and pharmacists	44	30.1
Implementation of the acts from Bill 41, established in 2015		
Totally (all acts and several adjustments)	50	34.2
Moderately (e.g., adjustment of anticoagulant therapy and some other)	59	40.4
Few (e.g., only anticoagulant therapy)	36	24.7
Not at all	1	0.7

Pharmacists were also asked to characterize the collaboration with other health care professionals. About one-third (36.3%) noted that professionals were available to answer calls from them, whereas 43.2% had difficulty reaching physicians. The number of pharmacists on each team varied from 4 to 6 for most respondents (57.5%). A third of pharmacists (30.1%) said vaccination was offered by both nurses and pharmacists in their work setting. When questioned about the implementation of activities allowed since 2015 (Bill 41), 40.4% of pharmacists said these activities were moderately implemented in their pharmacy (i.e., they adjusted anticoagulant therapy and some other pharmacotherapies). Even though these activities had been permitted for 6 years at the time of the survey, 25% of the respondents were still limiting their expanded scope of practice to anticoagulant adjustments or no activities.

### Comfort level and barriers in performing medication adjustments and follow-ups

The comfort level and barriers reported by the participating pharmacists for the 86 clinical situations are presented in Table 3. Pharmacists were comfortable performing follow-ups and medication adjustments in at least 1 of the situations presented for 9 of the 16 clinical conditions. Clinical situations in which almost all respondents were comfortable were adjustment for stable hypothyroidism (84.9%), type 2 DM with a single agent (84.9%), hypertension with a single agent (95.2%) or 2 to 3 antihypertensives (89.7%), primary and secondary cardiovascular prevention with statins (82.2% and 80.1%), anticoagulation with warfarin or direct oral anticoagulants, antidepressant withdrawal upon depression remission in patients without other psychiatric comorbidities (82.9%), management of a single antidepressant for anxiety disorders without any other psychiatric disorder (84.9%), gradual withdrawal of benzodiazepines and other hypnotics in insomnia (84.9%), adjustment of gabapentinoids in chronic pain (87.0%) and adjustment of psychostimulants in ADHD (children 80.8% and adults 84.2%). In situations in which almost all pharmacists were comfortable, most also reported no main barriers. The remaining participants mostly reported that lack of experience was their main barrier.

**Table 3:** Comfort level and barriers in performing adjustment of medication and follow-ups for different pathologies and complexity levels (*N* = 146)

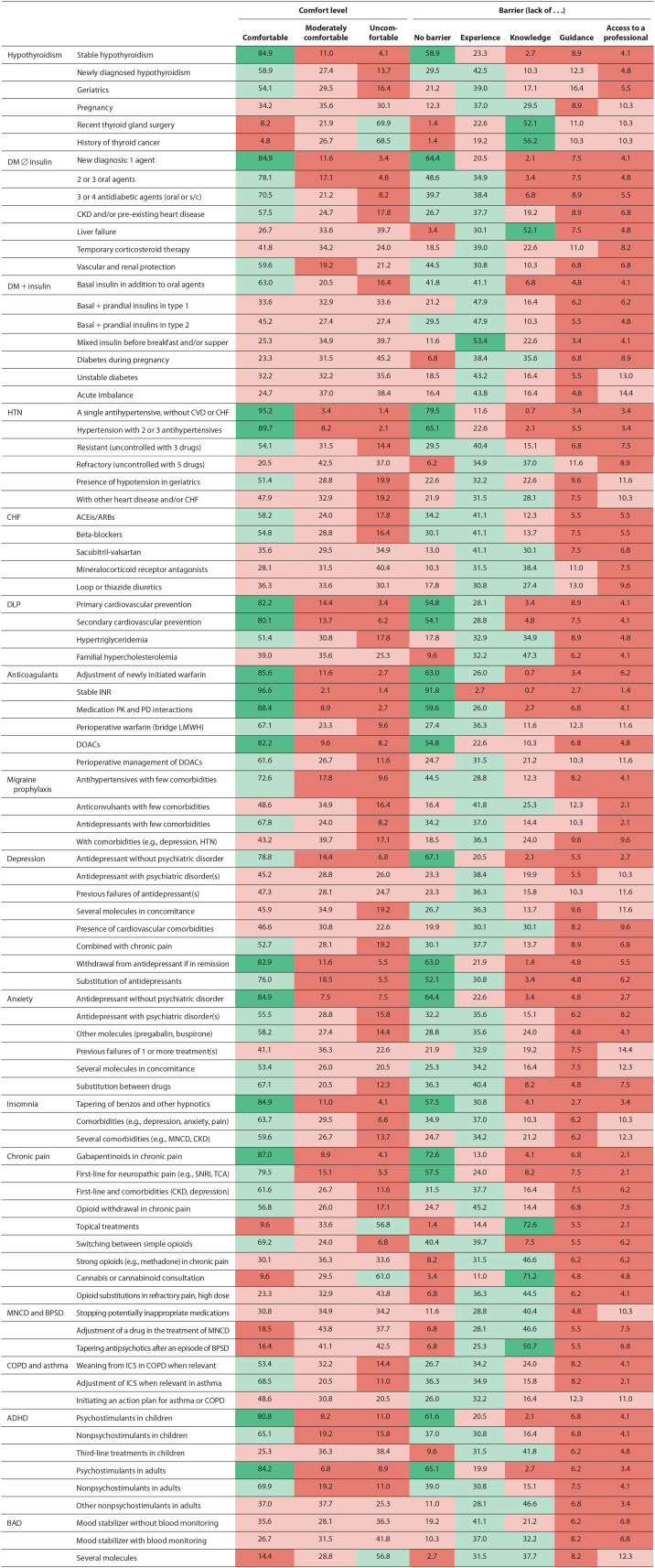

ACEis, angiotensin-converting enzyme inhibitors; ADHD, attention-deficit hyperactivity disorder; ARBs, angiotensin II receptor blockers; BAD, bipolar affective disorder; BPSD, behavioural and psychological symptoms of dementia; CHF, congestive heart failure; CKD, chronic kidney disease; COPD, chronic obstructive pulmonary disease; CVD, cardiovascular disease; DLP, dyslipidemia; DM, diabetes mellitus; DOACs, direct oral anticoagulants; HTN, hypertension; ICS, inhaled corticosteroids; INR, international normalized ratio; LMWH, low-molecular-weight heparin; MNCD, major neurocognitive disorder; PD, pharmacodynamics; PK, pharmacokinetics; s/c, subcutaneous; SNRI, serotonin and norepinephrine reuptake inhibitors; TCA, tricyclic antidepressant.Interest was a barrier only in cases in which pharmacists were not comfortable adjusting the medication (data not shown). Comfort levels are colour-coded when ≥80% (almost all respondents: dark green), >50% to <80% (most: light green), ≥20% to <50% (some: light pink) and less than 20% (very few: dark pink) reported feeling comfortable. Barriers are colour-coded when ≥50% of respondents reported no barrier (dark green), >25% to <50% (light green), ≥10% to <25% (light pink) and less than 10% (dark pink).

Clinical situations in which pharmacists were generally less comfortable were related to bipolar disorder, major neurocognitive disorder, behavioural and psychological symptoms of dementia, COPD and asthma. We identified 7 clinical situations in which very few (less than 20%) respondents were comfortable performing follow-ups and medication adjustments. Indeed, 69.9% and 68.5% of respondents claimed they were uncomfortable in managing hypothyroidism in patients with a recent thyroid gland surgery or a history of thyroid cancer, respectively. Other clinical situations in which many respondents felt uncomfortable were managing diabetes during pregnancy (45%), managing the use of topical pain treatments (56.8%), recommending cannabinoids (61%), tapering antipsychotics after a BPSD episode (42.5%) and managing bipolar disorder with several medications (56.8%). In these more complex situations, the lack of knowledge was the main barrier identified by most respondents.

Between 2.7% and 16.4% of pharmacists identified lack of clear guidance from the initial prescriber as their main barrier to performing modifications and adjustments for all conditions and situations. As for the lack of access to a professional to refer to in case of questions, between 1.4% and 14.4% of pharmacists reported it was their main barrier.

### Global barriers and facilitators

The participants were asked questions to identify up to 3 general barriers and facilitators or solutions to increase their involvement in therapy adjustment and follow-ups (Table 4). The lack of time dedicated to clinical interventions and follow-ups and the difficulty of integrating their expanded scope of practice in their workflow were the barriers reported by most respondents (67.8% for both). Lack of work organization and clinical tools and insufficient remuneration for these new activities were also identified as barriers by 28.1%, 26.7% and 27.4% of pharmacists, respectively. Only 3.4% of the respondents had no interest in providing services related to their expanded scope of practice. On average, respondents estimated that 44% of their expanded scope of practice activities were not performed due specifically to a lack of work organization, despite their ability and confidence to do them.

**Table 4:** General barriers and facilitators (*N* = 146)

	*n*	%
Barriers to adjustments and follow-up (several answers possible, maximum of 3 items)		
Lack of time dedicated to clinical interventions and follow-ups	99	67.8
Difficulty integrating an extended scope of practice into the work chain flow	99	67.8
Lack of organization regarding follow-ups and interventions carried out	41	28.1
Insufficient remuneration for the model to be viable	40	27.4
Lack of clinical tools or references to support practice	39	26.7
Lack of pharmacy technician training	20	13.7
Physicians’ lack of confidence to delegate clinical follow-ups to pharmacists	17	11.6
Lack of leadership from the pharmacy owner or head pharmacist	16	11.0
Different points of view and opinions between colleagues	15	10.3
Lack of knowledge about the new possibilities of Bill 31	14	9.6
I do not work many hours at the same pharmacy	6	4.1
Lack of interest in these new activities	5	3.4
Consequence of the lack of organization as a percentage of acts not performed despite ability and comfort (average: 44.0%)		
<20%	21	14.4
20% to <40%	31	21.2
40% to <60%	57	39.0
60% to <80%	18	12.3
80% and more	19	13.0
Solutions to implement more adjustments and follow-ups (several answers possible, maximum of 3 items)		
Improvement of the organization structure of my environment	79	54.1
Summary tables (summary of follow-up to be carried out)	74	50.7
Continuing education	56	38.4
Joint follow-up request form sent by the prescriber	33	22.6
Clinical follow-up documentation form carried out by the pharmacist	31	21.2
Discussion of complex cases with other pharmacists	28	19.2
Team discussion and meetings within the pharmacy team	26	17.8
Bank of examples of possible interventions	22	15.1
Consultation request form sent by the prescriber	19	13.0
Target request form to be sent to the prescriber	18	12.3
Access to frequently asked questions bank	11	7.5
Territorial meeting (local table or CRSP)	10	6.8
University level courses	6	4.1

CRSP, Comité régional sur les services pharmaceutiques.

Most respondents identified the improvement of work organization (54.1%) and the availability of follow-up summary tools (50.7%) as their main possible solutions. Continuing education was viewed as a facilitator by 38.4% of participants. The other identified facilitators were the ability to discuss complex cases with other pharmacists and the availability of validated clinical follow-up forms for communication between initial prescribers and pharmacists.

## Discussion

Our objective was to assess the comfort level of community pharmacists in adjusting medications and performing patient follow-ups after their scope of practice was expanded. We also aimed to identify barriers impeding the implementation of this expanded scope of practice to provide pharmacists with support in implementing these new activities. Among the clinical situations surveyed, we identified several in which most pharmacists were comfortable performing follow-ups and medication adjustments. These situations were generally either for first-line treatments or activities already allowed in the scope of practice. As expected, we observed no main barrier in situations in which most pharmacists felt comfortable. However, some pharmacists mentioned they still lacked the experience to feel comfortable in these simple situations.

Overall, pharmacists felt less comfortable with complex clinical situations. This was also observed by Makowsky et al.^6^ in Alberta, where pharmacists indicated they were less likely to prescribe in complex situations such as polypharmacy, unstable conditions, unclear diagnosis or beyond the scope of common clinical guidelines. A qualitative study among prescribing nurses and pharmacist prescribers in the United Kingdom also found some reluctance to take full prescribing responsibility for high-risk patients and when deviating from protocols and guidelines.^9^ Only 7 of 86 clinical situations had less than 20% of respondents feeling comfortable performing follow-ups and medication adjustments. Lack of knowledge was a common barrier in those more complex situations. Significant correlations between knowledge and experience, knowledge and confidence and experience and confidence were also observed in the literature for pharmacists performing patient-centred related activities.^5^ Thus, the more experience and knowledge pharmacists gain in a domain, the more confident they feel in being responsible for adjusting their patients’ therapy in similar situations. This suggests that to facilitate the implementation of expanded clinical activities, pharmacists must have access to continuing education to complement their knowledge and clinical training. Access to a more experienced mentor while they gain experience could also be beneficial, as suggested by other authors.^9-11^ This support could be provided by pharmacists practising in family medicine groups, who have access to the patient’s electronic medical record and are located in the primary care clinics and thus who have easier access to medical colleagues.^12^ Pharmacists practising in family medicine groups could offer their help, especially when transferring a patient follow-up to their colleagues.

Despite their ability and confidence to adjust medications in certain situations, pharmacists identified many barriers impeding their capacity to do so in their work setting, thus affecting the implementation of the new services. The 2 main identified barriers in our study were lack of time dedicated to follow-ups and difficulty integrating new activities into their workflow. Time constraints were also 1 of the major barriers to providing extended and enhanced pharmacy services reported by Australian pharmacists.^13^ In an American study published in 2016,^14^ pharmacists identified factors affecting collaborative practice agreement (CPA; in which the prescriber delegates the authority to perform specific patient care functions such as initiating, monitoring and adjusting drug therapies to a pharmacist) implementation in community pharmacies. They noted that implementation of CPAs has been limited in community settings because of financial, physical and policy barriers.^14^ Other barriers included lack of access to patient information, inadequate compensation and slow adoption and referral rate of patients from physicians.^14^ Lott et al.^15^ also studied barriers to CPA implementation in community pharmacies. They found that liability and billing issues, logistic concerns and a lack of information and resources to establish and maintain a CPA were major barriers to their implementation.^15^ These studies highlight the importance of organizational barriers to implementing expanded services in community pharmacies, which we also found in our study. They also suggest these new activities require pharmacists to work differently and therefore to adapt their environment and work organization to their new reality.

Certain models with physical adaptations to pharmacies and their work organization are being implemented. For instance, a banner recently opened dedicated clinical care spaces in pharmacies in Québec.^16^ These are equipped with a reception, waiting room and closed consultation rooms, giving patients access to dedicated pharmacists and a team of health care professionals.^16^ Having a private consultation area and space for a patient to lie down were positively associated with providing enhanced services^13^ and could improve patients’ acceptability of this new role for pharmacists.^17^

Some barriers were related to pharmacists’ or pharmacy technicians’ training and competencies. In fact, 11.6% of pharmacists mentioned the lack of confidence from physicians as a barrier to implementing their full scope of practice. Perceptions of the pharmacist’s role and a mistrust of pharmacists’ knowledge and competencies by physicians were also barriers to providing enhanced pharmacy services found in the literature from Canada, the United Kingdom, the United States and Australia.^9,11,18-20^ However, in our study, the proportion of respondents reporting this as a barrier was low, which might suggest that pharmacists’ competencies are generally recognized in Québec. The public perceptions of pharmacists’ knowledge and skills in providing expanded services in Canada have also been found to be positive.^21^ The fact that only 1 respondent reported no openness and collaboration from other health care professionals suggests that other clinicians are open to interprofessional collaboration and working with pharmacists to their full scope of practice. As for pharmacy technicians, 13.7% of respondents mentioned that their lack of training was a barrier. In February 2021, a college diploma for the training of pharmacy technicians was created in Québec.^22^ This diploma provides a higher level of knowledge of technical, administrative and clinical pharmaceutical tasks than the training for technical assistant in pharmacy (professional high school level). The first graduates are expected in 2024, and it is hoped that their increased training and knowledge will contribute to better supporting pharmacists in their expanded scope of practice.

One of the main facilitators found in our study was the availability of summary tables suggesting what should be followed up, when and targets to be reached. Since the survey was conducted, our team developed such evidence-based tools in collaboration with experienced pharmacists. The tools are offered through our practice-based network of pharmacists.^7^ The main facilitators for pharmacists’ involvement as independent prescribers found in the literature were conscious team-building efforts to establish trust and collaboration between physicians and pharmacists.^6,19^ Although it was not explicitly highlighted in our study, the roles of each professional in prescribing and adjusting medications have yet to be more clearly defined, with multiple professionals being able to intervene for the same patient. It is also important for the unique contribution of pharmacists to be understood by other health care providers so collaboration can occur, which is not always the case.^18^ These new activities enable pharmacists to play a more important role in patient follow-up, especially considering some citizens do not have a family doctor. However, as respondents have highlighted lack of time to perform follow-ups as a barrier, it will be important for them to identify which patients will benefit the most from their interventions specifically vs from another health care professional.

As the pharmacy profession evolves, the overall structural and organizational factors influencing pharmacy practice must be considered. Adaptation to the current environment is the key to success, and the capacity for organizational change can be augmented by increased proactiveness, autonomy among employees and the availability of appropriate and adequate resources.^23^ Several changes must be made to community pharmacies to transition from a dispensing-focused model to a clinically focused practice. For instance, automating or delegating more activities to technicians could decrease pharmacists’ workload and allow them to focus on clinical activities. This model is already in use in hospital pharmacies, and benefits have been seen in a study in which product verification was performed by a pharmacy technician in a community pharmacy in the United States.^24^ Indeed, time spent by pharmacists in clinical activities doubled after delegation, from 25.9% to 58.6%. As Dikun et al. highlighted in their study, “To support practice advancement, future efforts should account for both individual and environmental factors influencing the implementation process for new practices.”^25^

This study has several strengths. It is the first study that focuses on an in-depth understanding of how community pharmacists perceive their activities after further expansion of their scope of practice in Québec. The survey, which explored different aspects influencing the pharmacist’s comfort level with independently adjusting medications for several clinical situations, was pretested by experts before use and was designed to match the clinical activities allowed. However, we must also acknowledge some limitations of our survey. First, only 2% of community pharmacists in Québec completed the questionnaire. This low response rate could be explained by the length of the questionnaire and the time required for completion. Therefore, pharmacists who responded might not represent all pharmacists in Québec and could be more motivated and engaged in these new activities. Consequently, the comfort level measured in our study might be overestimated. In addition, the questionnaire was available for 2 months during the COVID-19 pandemic. It is possible that pharmacists were affected during this period by increased workload and time constraints, which influenced their assessment of barriers.^26^ Translating our results to other health care systems is difficult since pharmacists’ scope of practice varies in different provinces or countries. Nevertheless, the results showed barriers and facilitators that could translate to countries with similar contexts, and suggest ways to support the successful implementation of pharmacists’ expanded scope of practice and improve pharmacists’ confidence in accomplishing new clinical activities.

## Conclusion

This study shows that community pharmacists in the province of Québec are prepared to adjust pharmacotherapy and ensure follow-up in several clinical situations. However, they must have adequate time available and resources to support them. The more complex the clinical situations, the less comfortable community pharmacists feel in adjusting pharmacotherapy. Continuing education, training and mentoring could be offered in these areas since the main barriers were a lack of experience and knowledge. ■

## Supplemental Material

sj-pdf-1-cph-10.1177_17151635241264517 – Supplemental material for Community pharmacists’ comfort levels with and barriers to application of an expanded scope of practice in Québec Supplemental material, sj-pdf-1-cph-10.1177_17151635241264517 for Community pharmacists’ comfort levels with and barriers to application of an expanded scope of practice in Québec by Ĺonie Rouleau, Ĺa Prince-Duthel, Marie-Claude Vanier, Nicolas Dugŕ, Anne Maheu and Line Gúnette in Canadian Pharmacists Journal / Revue des Pharmaciens du Canada
