# Supplementary material for: Community pharmacists’ comfort levels with and barriers to application of an expanded scope of practice in Québec
Source: Can Pharm J (Ott). 2024 Jul 30;157(5):246–60. doi: 10.1177/17151635241264517 (PMC11412463; doi:10.1177/17151635241264517)
Supplement: sj-pdf-1-cph-10.1177_17151635241264517 – Supplemental material for Community pharmacists’ comfort levels with and barriers to application of an expanded scope of practice in Québec [file sj-pdf-1-cph-10.1177_17151635241264517.pdf]

APPENDIX 1 Supplementary materials

Questions (in French) that were included in the questionnaire.

DÉMOGRAPHIE (8 questions)
Quel est votre sexe ?
Féminin
Masculin

Dans quelle tranche d'âge vous situez-vous ?
Moins de 30 ans
30 à 39 ans
40 à 49 ans
50 à 59 ans
60 ans et plus

Quelles études avez-vous réalisées? (multiples choix)
Baccalauréat en pharmacie
Doctorat de premier cycle en pharmacie (PharmD)
Qualification en pharmacie (QeP)
Programme en développement du médicament (DESS)
Maîtrise en pharmacothérapie avancée : profil établissement de santé
Maîtrise en pharmacothérapie avancée : profil ambulatoire
DESS en pharmacie communautaire
DESS en pratique pharmaceutique de 1re ligne (programme passerelle)
Certificat de 2e cycle en pratique pharmaceutique de 1re ligne (programme passerelle pour le Pharm. D)
Microprogramme de 2e cycle en soins pharmaceutiques
Microprogramme en pharmacie communautaire
Microprogramme en gestion de pharmacie
Autre, veuillez préciser : ___________________________

En quelle année avez-vous obtenu votre permis de pratique de la pharmacie au Québec ?

Combien d'années d'expérience comme pharmacien avez-vous dans chacun de ces milieux ?
Années
- Aucune
- < 6 mois
- 6 à 12 mois
- > 1 an à 2 ans
- > 2 ans à 5 ans
- > 5 ans à 10 ans
- > 10 ans

Milieux
- GMF :

- Communautaire :
- Établissement de santé :
- Enseignement (Cégep, Université)
- Autre : Veuillez préciser : _______________

Dans quelle région administrative du Québec se trouve votre lieu d'exercice principal ? Pour les régions de Montréal et de la Montérégie, sélectionnez votre CISSS/CIUSSS de pratique.

- 01-Bas St-Laurent
- 02-Saguenay-Lac-St-Jean
- 03-Capitale-Nationale
- 04-Mauricie-et-du-Centre-du-Québec
- 05-Estrie
- 06-Montréal - Nord-de-l'Ile
- 06-Montréal - Centre Sud-de-l'Ile
- 06-Montréal - Centre Ouest-de-l'Ile
- 06-Montréal - Est-de-l'Ile
- 06-Montréal - Ouest-de-l'Ile
- 07-Outaouais
- 08-Abitibi-Témiscamingue
- 09-Côte-Nord
- 10-Nord-du-Québec
- 11-Gaspésie-Îles-de-la-Madeleine
- 12-Chaudières-Appalaches
- 13-Laval
- 14-Lanaudière
- 15-Laurentides
- 16-Montérégie-Est
- 16-Montérégie-Centre
- 16-Montérégie-Ouest

À quel titre exercez-vous principalement la pharmacie en communautaire ?

- Pharmacien salarié (temps plein)
- Pharmacien salarié dans un milieu (temps partiel)
- Pharmacien salarié dans plusieurs milieux fixes
- Pharmacien remplaçant dans divers milieux
- Pharmacien chef
- Pharmacien propriétaire

Travaillez-vous actuellement dans un autre milieu (plusieurs choix possibles) ?
Oui, en établissement de santé (hôpital)
Oui, en établissement de santé (autre qu'hôpital)
Oui, en enseignement (CÉGEP, université)
Oui, en GMF
Non, je travaille uniquement en communautaire
Autre, veuillez préciser : _______________

ENVIRONNEMENT ET ORGANISATION DU TRAVAIL (9 questions)

Dans quelle chaîne ou bannière travaillez-vous principalement actuellement ?

- Accès pharma
- Brunet
- Costco
- Familiprix
- Groupe Horizon Santé
- Jean Coutu
- Pharmaprix
- Proxim
- Uniprix
- Pharmacie indépendante
- Autre

Combien de pharmaciens composent votre équipe (nombre de pharmacien total, incluant le propriétaire s'il travaille dans le laboratoire)

Du lundi au vendredi, quel est le nombre moyen d'ordonnances par heure, par pharmacien? (incluant prescriptions, renouvellements, piluliers, nouveaux actes, etc.)
Exemple: Du lundi au vendredi, vous faites 300 Rx en moyenne par jour et vous êtes ouvert de 9h00 à 21h00. Le pharmacien est doublé 8 heures par jour, excluant les pauses-repas. 300 Rx/20 h phm = 15 Rx/h/phm

- 50 et plus
- 40-50
- 30-40
- 20-30
- 10-20
- < 10
- je ne sais pas

La délégation de la vérification contenant-contenu (DVCC) est-elle en place ?

1. Oui pour les piluliers et les paniers de renouvellement
2. Oui seulement pour les piluliers
3. Oui, partiellement ou sur une base occasionnelle
4. Pas du tout

Est-ce qu'une plage horaire est dédiée aux suivis cliniques ?

- Oui nous avons un pharmacien et une plage horaire planifiée à chaque jour
- Oui nous avons un pharmacien et une plage horaire planifiée certains jours
- Nous n'avons pas de plage horaire dédiée mais il est convenu qu'un pharmacien le fasse lorsqu'il est doublé.
- Non

En moyenne, relativement au total de vos heures travaillées, quel pourcentage de votre temps de travail est réservé aux suivis cliniques ? (écrivez le % entre 0 et 100)

De façon générale, comment décririez-vous la collaboration interprofessionnelle avec les professionnels pratiquant dans votre région ?

- Grande ouverture à la collaboration de part et d’autre
- Professionnels disponibles pour répondre aux appels des pharmaciens
- Difficile de rejoindre les médecins mais certaine collaboration possible
- Aucune ouverte à la collaboration
- Autre :

<u>Offrez-vous le service de vaccination ? (plusieurs réponses)</u>
Types de vaccinateurs
- Aucun
- Infirmiers(ères)
- Pharmaciens(nes)
- Infirmiers(ères) et Pharmaciens(nes)

Types
- Vaccins usuels
- Vaccination de masse (influenza et COVID-19).

<u>Aviez-vous déjà implanté les actes de prise en charge de la loi 41?</u>
1. Totalement (tous les actes et plusieurs ajustements)
2. Modérément (p. ex. ajustement de l’anticoagulothérapie et quelques autres pathologies)
3. Peu (p. ex. seulement l’anticoagulothérapie)
4. Pas du tout

<u>AISANCE ET OBSTACLES</u>

<u>Mise en contexte (2 questions x 87 sous-groupes = 174 questions)</u>
Voici des mises en situations pour différentes pathologies où le pharmacien peut prendre en charge l’AJUSTEMENT et le SUIVI des patients avec les nouvelles activités de la loi 31.

Question 1 : Pour chaque situation suivante, indiquez si vous vous sentez à l’aise d’effectuer ce suivi et ajustement
- Totalement à l'aise : Vous jugez que vous pourriez le réaliser dans tous les cas
- Assez à l’aise : Vous auriez besoin d’un peu de temps pour le réaliser ou besoin de gagner en expérience, mais dans la majorité des cas vous seriez à l’aise de le faire
- Moyennement à l’aise : Vous auriez besoin de consulter des références plus approfondies, un collègue ou un spécialiste. Vous auriez besoin de développer vos connaissances. Ça pourrait aussi être dans certaines circonstances, si on vous indiquait des moments de suivis, des doses maximales de titration et/ou des cibles (p.ex : TA, glycémies, nombre de migraine, ….)
- Peu à l’aise : Vous manquez de connaissances, de compétences et/ou d’informations pour réaliser ces prises en charge. Vous vous sentez peu habileté à faire des ajustements et suivis de par la présence de plusieurs obstacles (connaissance, compétence, habiletés, ...).
- Pas du tout à l’aise : Vous sentez que c’est une situation qui dépasse l’étendue de ce que vous seriez à l’aise de faire comme pharmacien.

Question 2 : Pour chaque situation suivante, indiquez l’obstacle PRINCIPAL à ce que vous vous sentiez à l’aise (sentiment de confiance) d’effectuer les suivis et ajustements. Nous voulons

connaître ce qui vous rendrait plus apte à réaliser ces prises en charge, en supposant qu'elles vous soient déléguées par le professionnel (MD ou IPS). Nous sommes conscients que l'organisation du travail peut être un obstacle majeur à la prise en charge de patients, mais dans cette question nous désirons plutôt connaître les obstacles à ce que vous soyez confortable au niveau clinique à réaliser les prises en charge, soit l'ajustement et le suivi des patients.

- Aucun obstacle
- Manque d'expérience clinique pour l'effectuer avec assurance
- Manque de connaissances sur le sujet
- Manque d'accès à un professionnel à qui me référer en cas de questions
- Manque de directive claire (cibles, suivis et doses max, communication attendue)
- Manque d'intérêt

<u>Situations (87 sous-groupes)</u>

Hypothyroïdie (6 sous-groupes)

- Hypothyroïdie stable (euthyroïdie depuis des années et bonne adhésion au traitement)
- Hypothyroïdie nouvellement diagnostiquée
- Hypothyroïdie en gériatrie
- Hypothyroïdie durant la grossesse
- Chirurgie récente de la glande thyroïde
- Antécédents de cancer de la thyroïde

Diabète non insulinodépendant (7 sous-groupes)

- Nouvellement diagnostiqué avec un seul agent antihyperglycémiant
- Diabète avec 2 ou 3 agents antihyperglycémiants per os
- Diabète avec 3 ou 4 agents antidiabétiques incluant un agoniste GLP-1
- Diabète en contexte de IRC et/ou MCV déjà installées
- Diabète avec insuffisance hépatique
- Sous corticothérapie de manière temporaire
- Amorcer une protection vasculaire et rénale (p. ex. l'ajout d'un IECA/ARA ou d'une statine)

Diabète insulinodépendant (7 sous-groupes)

- Insuline basale (seule) en ajout aux agents antidiabétiques oraux
- Insulines basale + prandiales en DMT1
- Insulines basale + prandiales pour en DMT2
- Insuline mixte avant le déjeuner et/ou avant le souper
- Diabète durant la grossesse
- Débalancement fréquent des glycémies nécessitant un suivi rapproché
- Débalancement aigu nécessitant un suivi rapproché

Hypertension (6 sous-groupes)

- Un seul antihypertenseur, sans MCV ni IC.
- 2 ou 3 antihypertenseurs
- Hypertension résistante (non contrôlée avec 3 médicaments)
- Hypertension réfractaire (non contrôlée avec 5 médicaments)
- Présence d'hypotension en gériatrie
- Avec autres MCV et/ou IC

Insuffisance cardiaque (5 sous-groupes)

- Titration des IECA/ARA jusqu'à la dose cible ou dose maximale tolérée
- Titration des Bêta-bloqueurs jusqu'à la dose cible ou dose maximale tolérée
- Titration de sacubitril-valsartan
- Ajustement d'antagonistes des récepteurs minéralocorticoïdes
- Ajustement de diurétiques de l'anse ou thiazidiques

Dyslipidémie (4 sous-groupes)

- Prévention cardiovasculaire primaire
- Prévention cardiovasculaire secondaire
- Hypertriglycéridémie
- Hypercholestérolémie familiale

Anticoagulation (6 sous-groupes)

- Ajustement de la warfarine nouvellement débutée
- Suivi du RNI et ajustement de la warfarine chez la personne ayant un RNI stable
- Ajustement de la warfarine en présence d'interactions
- Gestion de la warfarine en péri-opératoire (p. ex. considérer pont HFPM)
- Ajustement de dose des anticoagulants oraux directs (AOD)
- Gestion des AOD en péri-opératoire

Prophylaxie de la migraine (4 sous-groupes)

- Peu de comorbidités, traité avec les antihypertenseurs
- Peu de comorbidités, traité avec les anticonvulsivants
- Peu de comorbidités, traité avec les antidépresseurs
- Avec présence de plusieurs comorbidités (p. ex. dépression, hypertension, ...)

Trouble dépressif (8 sous-groupes)

- Un premier antidépresseur sans autre trouble psychiatrique
- Un premier antidépresseur avec d'autre(s) trouble(s) psychiatrique(s)
- Échecs antérieurs à un ou plusieurs antidépresseur(s)
- Plusieurs molécules en concomitance (antidépresseur ou autres)
- Avec présence de comorbidités cardiovasculaires
- Avec également de la douleur chronique
- Sevrage d'un antidépresseur chez la personne en rémission depuis au moins 6 mois
- Substitution d'antidépresseurs de même classe ou de classes différentes

Anxiété (6 sous-groupes)

- Un seul antidépresseur sans autre trouble psychiatrique
- Un premier antidépresseur avec d'autre(s) trouble(s) psychiatrique(s)
- Un seul médicament autre que les antidépresseurs (p. ex. prégabaline, buspirone)
- Échecs antérieurs à un ou plusieurs traitement(s)
- Plusieurs molécules en concomitance (antidépresseur ou autres)
- Substitution entre deux médicaments de même classe ou de classes différentes

Insomnie (3 sous-groupes)

- Sevrage progressif des benzodiazépines et autres hypnotiques
- Présence de certaines comorbidités associées (p. ex. dépression, anxiété, douleur chronique)
- Présence de plusieurs comorbidités (TNC, IRC, ...)

Douleur chronique (10 sous-groupes)

- Ajustement de prégabaline ou de gabapentine
- Ajustement des traitements oraux de première intention en douleurs neuropathiques (p. ex. IRSN, antidépresseurs tricycliques)
- Ajustement des traitements oraux de première intention en douleurs neuropathiques (p. ex. IRSN, antidépresseurs tricycliques) avec présence de plusieurs comorbidités (IRC, gériatrie, dépression)
- Sevrage d’opioïde
- Consultation sur les traitements topiques (p. ex. kétamine, lidocaïne, amitriptyline)
- Consultation sur les changements entre opioïdes simples (p. ex. morphine)
- Consultation sur les opioïdes puissants (p. ex. méthadone)
- Consultation sur le cannabis ou les cannabinoïdes
- Consultation sur les substitutions d'opioïdes en cas de douleurs réfractaires aux hautes doses d'opioïdes

Troubles neurocognitifs majeurs (TNCM) et symptômes comportementaux et psychologiques de la démence (SCPD) (3 sous-groupes)

- Sevrage (ou refus d’initier) des médicaments potentiellement inappropriés en TNC
- Ajustement d'un médicament dans le traitement des TNCM
- Sevrage des médicaments (antipsychotiques) suite à un épisode aigu de SCPD

MPOC et asthme (3 sous-groupes)

- Sevrage des CSI en MPOC lorsque pertinent
- Ajustement de la dose de CSI lorsque pertinent en asthme (stable ou non contrôlé)
- Amorcer un plan d’action (lorsque non prescrit préalablement)

TDAH enfant (3 sous-groupes)

- Psychostimulants dans le cadre d’une prescription valide avec plusieurs étapes
- Non psychostimulants (guanfacine et atomoxétine)
- Traitements de 3e ligne (clonidine, rispéridone)

TDAH adulte (3 sous-groupes)

- Psychostimulants dans le cadre d’une prescription valide avec plusieurs étapes
- Non psychostimulants (atomoxétine)
- Autres traitements non psychostimulants (bupropion, modafinil, ...)

Trouble bipolaire (3 sous-groupes)

- Stabilisateur de l’humeur ne nécessitant pas de suivis sanguins rapprochés (p. ex. lamotrigine)
- Un seul stabilisateur de l’humeur (lithium, divalproex) requérant un suivi sanguin (concentration plasmatique, hématologique, métabolique) avec peu d’échec antérieur
- Plusieurs molécules (stabilisateurs et/ou antipsychotique)

OBSTACLES ET SOLUTIONS (3 questions)

Selon vous, quels sont les obstacles principaux à la prise en charge, aux ajustements et suivis dans votre milieu de pratique ? (plusieurs réponses possibles, choisir maximum 3 items)

- Je ne travaille pas beaucoup d'heures à la même pharmacie.
- Vision divergente entre collègues pharmaciens pour ce qui est des nouveaux actes.
- Équipe trop nombreuse
- Manque de leadership du propriétaire ou du pharmacien chef
- Manque d'organisation dans le suivi et les interventions à effectuer
- Manque de temps dédié aux interventions et suivis cliniques
- Manque d'intérêt envers ces activités
- Intégration difficile dans le flux de la chaîne de travail
- Manque d'outils cliniques ou méconnaissance des références pour supporter la pratique
- Insuffisance de la rémunération pour que le modèle soit viable
- Manque de connaissance sur les nouvelles possibilités de la loi 31
- Manque de confiance de la part des médecins pour déléguer les suivis
- Manque de formation des ATP
- Autre

L'organisation du travail dans votre milieu est un obstacle important à la réalisation des suivis cliniques par le pharmacien. Selon vous, quel est le pourcentage d'actes que vous ne réalisez pas, à cause de l'organisation du travail et du temps (non-dédié), malgré votre compétence à les faire ? (écrivez le % entre 0 et 100)
100% = je ne réalise aucune acte pour lesquels je me sens compétent à cause de l'organisation du travail
50% = je suis en mesure de réaliser seulement 50% des actes pour lesquels je me serais senti à l'aise à cause de l'organisation du travail
0%= je réalise tous les actes pour lesquels je me sens compétent

De quoi auriez-vous besoin pour mieux implanter ou mieux faire vos interventions et suivis cliniques ? (plusieurs réponses possibles, choisir maximum 3 items)

- Formulaire de demande de cibles à envoyer au MD
- Formulaire de demande de suivi conjoint envoyé par le MD
- Formulaire de demande de consultation envoyé par le MD
- Formulaire de documentation du suivi clinique réalisé par le pharmacien
- Formation continue
- Tableaux récapitulatifs (synthèse des suivis à effectuer)
- Discussion d'équipe au sein de la pharmacie
- Amélioration de la structure organisationnelle de mon milieu
- Rencontre territoriale (table locale ou CRSP)
- Discussion de cas complexes avec d'autres pharmaciens
- Cours de niveau universitaire
- Accès à une foire aux questions les plus fréquemment posées
- Banque d'exemples d'intervention possibles
- Autre :

Rouleau L, et al. Community pharmacists' comfort levels with and barriers to application of an expanded scope of practice in Quebec. *Can Pharm J (Ott)* 2024;157. DOI: 10.1177/17151635231264517.
